# Formation of Silicide and Silicide-Aluminide Coatings on Molybdenum Alloy during Slurry Cementation Process: Influence of Slurry Volume

**DOI:** 10.3390/ma14226940

**Published:** 2021-11-17

**Authors:** Agnieszka Elżbieta Kochmańska, Aneta Jarlaczyńska, Jolanta Baranowska

**Affiliations:** Faculty of Mechanical Engineering and Mechatronics, West Pomeranian University of Technology in Szczecin, Av. Piastow 17, 70-310 Szczecin, Poland; aneta.jarlaczynska@zut.edu.pl (A.J.); Jolanta.Baranowska@zut.edu.pl (J.B.)

**Keywords:** slurry method, silicide coatings, aluminide coatings, molybdenum alloy

## Abstract

New slurry cementation method was used to produce silicide and silicide-aluminide protective coatings on molybdenum alloy (TZM). The slurry cementation processes were carried out at a temperature of 1000 °C in different time intervals with the use of varied slurry mass values. The microstructure and thickness of the coatings were studied by means of scanning microscopy. Chemical composition using X-ray microanalysis and phase composition using X-ray diffraction were also investigated. Coating microhardness was determined. The obtained coatings had a multilayer structure. Phases from the Al-Si-Mo system were observed in silicide-aluminide coatings and phases from the Si-Mo system were observed in silicide coatings. The microhardness strongly depended on the phase composition of the coating. It was demonstrated that slurry mass values had an important influence on the morphology and growth kinetics of silicide-aluminide coatings. In the case of a small amount of the slurry, the deficiency of alloying elements occurring during long processes reduces growth kinetics and can lead to void formation in the structure of silicide-aluminide coatings.

## 1. Introduction

Molybdenum alloy TZM is a promising structural material for the aerospace industry and nuclear or conventional power plant applications owing to its high creep and yield strength at elevated temperature, excellent corrosion resistance against irradiation embrittlement beyond 800 °C, and good compatibility with alkali liquid metals [1]. In comparison with other metals used in these applications, such as titanium, niobium, molybdenum, tantalum, tungsten, and their alloys or creep-resistant steels and superalloys, TZM demonstrates the best combination of high allowed working temperature (1700 °C) and density (10.16 g/cm^3^). This material is the most suitable for use under high temperature and non-oxidizing conditions [1]. However, despite its high melting temperature and good mechanical properties, the use of TZM alloy at elevated temperature is limited by its poor resistance to oxidation. The MoO_3_ oxide that is formed on the TZM alloy surface in an oxidizing environment at 540 °C evaporates at 790 °C [2]. Therefore, at such temperatures, TZM alloy would have to be used in a controlled atmosphere such as vacuum or an inert gas, which is impractical in all the above-mentioned applications.

The formation of a protective coating on the alloy surface seems to be a good approach for improving the high temperature oxidation resistance of TZM alloy. An important requirement for such a coating is that it should ensure the formation of a self-healing protective oxide layer on the surface under working conditions, and in this way protect the TZM alloy against direct contact with atmospheric oxygen. Several coatings, mainly based on Cr, Ni, Au, B, Si, or Al, have already been studied to protect molybdenum alloys [3,4]. Of these, silicide coatings are considered to be the most attractive material for the protection of Mo and Mo-based alloys used in an oxidizing atmosphere at high temperature [3]. Such coatings usually contain the MoSi_2_ phase, which ensures the formation of a well-adherent and continuous SiO_2_ passive layer on the coated surface during high temperature oxidation. Furthermore, MoSi_2_ has a high melting temperature (2030 °C), excellent corrosion resistance at high temperature and moderate density (6.31 g/cm^3^). MoSi_2_ is also considered as an attractive material for high-temperature structural applications at high temperature, because of its compatibility with many ceramic reinforcements. The main drawbacks of coatings containing MoSi_2_ phase include a brittleness at temperatures up to 1000 °C and a strong tendency to pesting within the temperature range of 300–600 °C, due to the significant volume change of the MoSi_2_ phase (+85.5 vol.%) during oxidation. The latter issue can be solved by the addition of aluminium to the coating. This results in a silicide-aluminide coating containing Mo(Si,Al)_2_ phase, which demonstrates much smaller volume changes upon oxidation (+4.9 vol.%). This phase is also very important from the viewpoint of resistance to high temperature oxidation, as it promotes the formation of a thin protective Al_2_O_3_ layer on the coating surface [5].

Several techniques, particularly pack cementation [5,6,7,8], chemical vapor deposition [9], electrodeposition [10], and hot dip plating [11] have been used for the manufacture of silicide coatings on molybdenum and TZM alloy. All of the coatings obtained by these methods consist of MoSi_2_, Mo_5_Si_3_, and Mo_3_Si sub-layers [5,12]. This means that all the phases that should be formed according to the Mo–Si diagram system are present [13]. Pack cementation is the most common method used for the manufacture of aluminide coatings [14,15]. There is only limited information available in the literature regarding silicide-aluminide coatings produced on TZM alloy [12] and it is reported that the coatings obtained consist of Mo(Si,Al)_2_, Mo_5_(Si,Al)_3_, and Mo_3_(Si,Al) sub-layers [13,16].

The powder mixture, which is used in the pack cementation method for the production of aluminide coatings, contains very fine aluminum and silicon powders (with typical size of a few micrometers), aluminum oxide powder as a filler, and halides powder as an activator. The presence of aluminum oxide significantly reduces the thermal conductivity of the mixture. For this reason, the coating process should be either conducted at high temperature—at least 1000 °C or followed by an additional annealing at this temperature in order to obtain the desired coating composition and morphology [12]. The use of fine-grained aluminum powder in this process creates the risk of explosion, which is why special safeguards in the production process are required [6,12,17]. Moreover, a large amount of chloride and hydrogen chloride is emitted in the pack cementation process, which makes this method environmentally harmful [14,17]. Aluminide coatings can also be obtained by chemical vapor deposition; however, this method is economically unfeasible [12].

Slurry methods are an interesting alternative to pack cementation and CVD processes. These technologies provide easy manufacturing of coatings on complex-shaped parts at low cost and slurries that can be used for this purpose are commercially available. However, the organic binder contained in these slurries means that an additional annealing step is needed in production, in order to expel the binder from the slurry before the main coating manufacturing process can be carried out [18,19,20].

A new technological approach is proposed in this paper. Our innovative method is based on a slurry in which the organic binder has been replaced by sodium silicate (water glass), which allows the production of coatings in a single technological step. The slurry after drying has a good mechanical durability due to the properties of water glass, which is important from the technological point of view. A workpiece with the dried slurry can be transferred to the furnace to create a coating without a risk of damage. The presence of water glass in the slurry and additionally silicon is also very important, since the curing of the slurry proceeds due to the dehydration of sodium silicate (water glass) in the presence of silicon. The use of silicon powder is therefore important from the technological point of view during the application of the slurry and during the formation of a diffusion coating (co-introduction silicon into aluminide). At the same time, dehydration of the aqueous sodium silicate solution in the presence of salts (which are also present in the slurry) leads to the formation of a spatial gel network. This promotes the formation of a slurry with a suitable consistency (gel) that facilitates application.

The usefulness of this method for the production of both silicide and silicide-aluminide coatings on molybdenum alloy has been confirmed in previous works [21,22,23,24]. This slurry method can be applicable for materials of a raw cast surface thanks to the presence of a flux in the slurry [21]. It is possible to produce aluminide coatings on titanium and nickel alloys [23,24]. It is also possible to produce a silicide coating on molybdenum alloy composed of three sub-layers with a phase composition according to the Mo–Si diagram system [22]. The silicide-aluminide layers produced by this method have a multi-layered morphology as with other methods, but the phases obtained are much richer in aluminum than those presented in the literature manufactured using other methods. Importantly, the critical Mo(Si,Al)_2_ phase was present in all of the coatings obtained. The increased aluminum content measured in these phases can be beneficial from the viewpoint of corrosion resistance, ensuring sufficient aluminum supply during the self-healing process upon high temperature oxidation. The paper presents studies on the influence of the slurry volume used during the manufacturing process, on silicide and silicide-aluminide coating formation on TZM alloy.

## 2. Materials and Methods

Samples of dimensions 10 × 6 × 30 mm were prepared from TZM alloy containing approximately (% wt.): 0.5% Ti, 0.1% Zr, 0.02% C and Mo balance. The samples were mechanically grounded and cleaned in an acetone bath before being immersed in the slurry. Two types of slurries were prepared. The first slurry contained only silicon powder and the second a mixture of aluminum and silicon (with Al/Si mass ratio 9:1); these being the active components of the slurry used to obtain silicide or silicide-aluminide coatings, respectively. The grain size of the powder used was approximately 100 μm for aluminum and within the range 200–250 μm for silicon. The further composition of the slurries was the same for both processes, with an inorganic binder (an aqueous solution of sodium silicate) and a flux (a mixture of fused sodium and potassium salts: NaF, NaCl, and KCl). The composition of the slurry was established experimentally. The ratio of the sodium silicate to powders of aluminum and silicon and the ratio of the salts to powders was 15/100 and 9.5/100, respectively.

The samples were immersed in the appropriate slurry and subsequently dried. The immersing and the drying operations were repeated several times where necessary until the slurry mass value achieved the appropriate amount. In the experiments three different quantities of slurries were applied: 0.15, 0.3, and 0.6 g/cm^2^, corresponding to one, two or four immersion steps, respectively. The resulting values were calculated based on weight measurements of the samples.

The samples, covered with the slurry, were annealed in an argon atmosphere at 1000 °C for 2, 4, and 6 h. After annealing, the slurry residues were mechanically removed, and the samples were washed in an ultrasonic bath in acetone. The residues easily detached from the surface after annealing.

The microstructure of the coatings was examined on cross-sections using field emission scanning electron microscopy (FE-SEM)—Hitachi SU-70 (Hitachi, Naka, Japan) with X-ray microanalysis energy dispersive spectrometry (EDS)—Thermo Scientific NORAN System 7 (Madison, WI, USA), acceleration voltage 15 kV. X-ray diffraction (XRD) phase analysis was performed using Cu-Kα, using X-ray tube parameters of 35 kV and 45 mA and a Bragg–Brentano geometry (X’Pert–PRO, Panalytical, Almelo, The Netherlands). The applied step of the goniometer was 0.05°, and the acquisition time was 200 s. The acquired data were processed using X’Pert HighScore (v. 2.2.1) software provided by Panalytical. Microhardness tests were performed using a Buehler hardness tester MicroMet 2000 (Lake Bluff, IL, USA) with a Knoop indenter at a load of 0.098 N for 15 s. Microhardness tests were conducted on cross-sections of the coatings.

## 3. Results

### 3.1. General Coating Morphology

**Figure 1:**
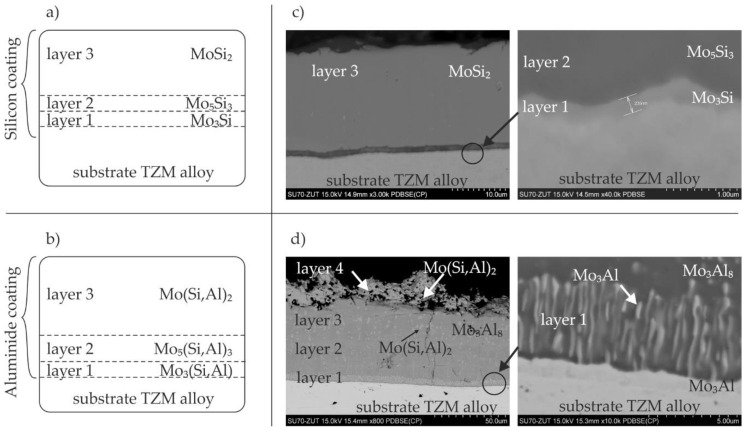
Graphical representation of (**a**) Si and (**b**) Si-Al coatings manufactured by the pack cementation method [5,6], and the microstructure of (**c**) Si coating and (**d**) Si-Al coating obtained by the slurry cementation process in the current studies (2 h at 1000 °C, slurry volume of 0.3 g/cm^2^).

As has been already mentioned, according to literature studies [5,6], silicide and silicide-aluminide coatings obtained on TZM alloys by the pack cementation method both consist of three sub-layers. Schematic representations of the coating morphology are presented in Figure 1a,b.

It was demonstrated in our previous studies [22] that our method is able to produce silicide coatings composed of three sub-layers with the same phase composition as the coatings obtained by other methods. Figure 1c shows an example cross-section of such a coating. The external sub-layer 3 was composed of the MoSi_2_ phase, which is the richest in silicon and accounts for almost 90% of the coating volume. This is because of the growth rate, higher for the MoSi_2_ phase than for the Mo_5_Si_3_ phase forming sub-layer 2 [25,26]. The Mo_3_Si phase, which is the richest in molybdenum and was approximately 200–300 nm thick, was formed adjacent to the substrate.

**Figure 2:**
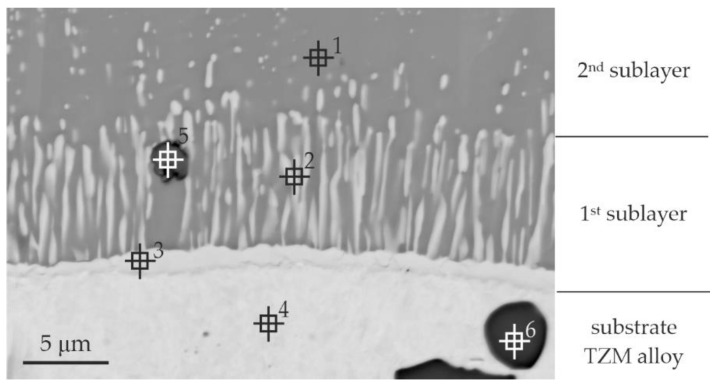
Microstructure (BEI) of a part of Si-Al coating (from the second group of samples) obtained on TZM alloy at temperature 1000 °C in 6 h with a quantity of slurry 0.3 g/cm^2^ with points of X-ray microanalysis.

The silicide-aluminide coatings produced by the pack cementation method are composed of Mo(Si,Al)_2_, Mo_5_(Si,Al)_3_, and Mo_3_(Si,Al) sub-layers as schematically shown in Figure 1b [5]. The silicide-aluminide layers produced by the method used in the current study also had a multi-layered morphology, but four sub-layers could be distinguished instead of the three observed in the coatings presented in the literature manufactured using the other method [5]. Importantly, the Mo(Si,Al)_2_ phase—fourth sub-layer, critical from the corrosion resistance point of view, was present on the top of all the coatings obtained. Sub-layers 3 and 2 were mainly composed of Mo_3_Al_8_ phase (point 1 in Figure 2 and Table 1) and MoAl_3_ phase, respectively. Moreover, numerous large, spherical precipitates of Mo(Si,Al)_2_ phase were spread throughout these sub-layers. Sub-layer 1 had a multiphase microstructure, i.e., elongated precipitates of Mo_3_(Si,Al) phase were uniformly distributed within a Mo_3_Al_8_ matrix (point 2 in Figure 2 and Table 1) and were oriented perpendicular to the sub-layer boundary. Mo_3_(Si,Al) phase also formed a continuous layer near to the substrate (point 3 in Figure 2 and Table 1). The sub-layers from 1 to 3 mostly contained Al, Mo-based phases, and were much richer in aluminum than the corresponding phases observed in other coatings (Figure 1b). The increased aluminum content measured in these phases can be beneficial from the viewpoint of corrosion resistance, ensuring sufficient aluminum supply in a self-healing process upon high temperature oxidation [27].

Additionally, titanium and zirconium carbides, which may be present in the alloy structure, were observed (points 5, 6 in Figure 2 and Table 1). It should be emphasized here that these precipitations were observed not only in the substrate, but also in sub-layers located next to the substrate (sub-layer 1 and 2).

**Figure 3:**
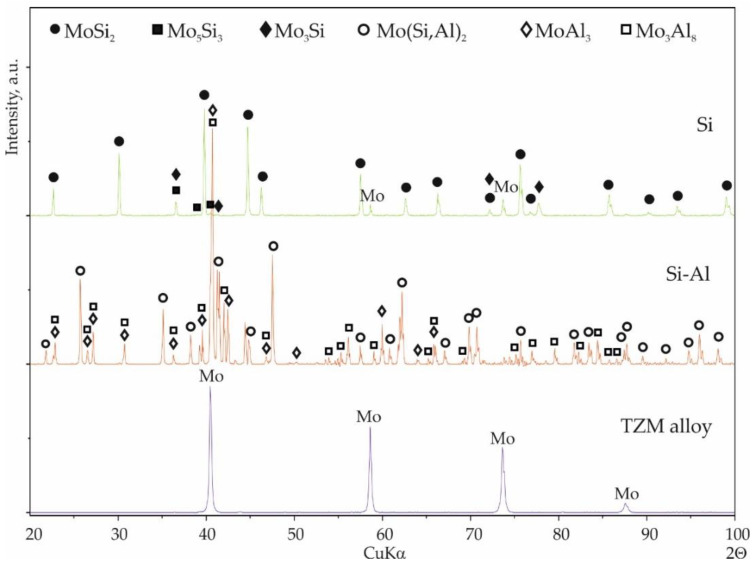
XRD patterns of Si and Si-Al coating obtained on TZM alloy at temperature 1000 °C in 2 h with a quantity of slurry 0.3 g/cm^2^.

XRD phase analysis was performed on the surfaces of all the coatings. The phase composition of the silicide and silicide-aluminide coatings is shown in Figure 3. Because the results were very similar for each coating group, one representative diffractogram pattern of each group is presented.

In the case of the silicide coatings, a clear presence of the α-MoSi_2_ phase was observed. In addition, weaker reflections from the Mo_5_Si_3_ and Mo_3_Si phases were also noticeable.

**Figure 4:**
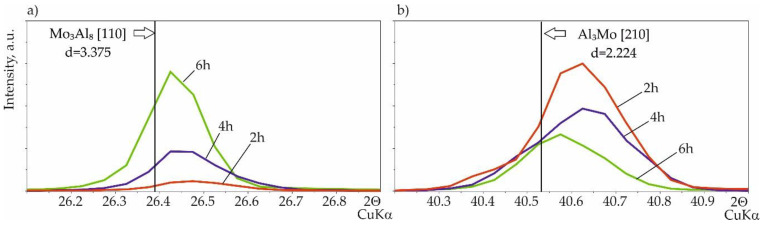
XRD pattern of Si-Al coating obtained on TZM alloy with a quantity of slurry 0.15 g/cm^2^ at temperature 1000 °C in 2 h (red color), 4 h (blue color), 6 h (green color); (**a**) the part within the angle range from 26.1 to 26.9; (**b**) the part within the angle range from 40.2 to 41.0.

After analyzing the diffractogram pattern of the silicide-aluminide coatings, the phases Mo(Si,Al)_2_, Mo_3_Al_8_, and MoAl_3_ were identified. Some differences were found in the diffraction patterns obtained for the silicide-aluminide coatings, mainly regarding the intensity of the MoAl_3_ and Mo_3_Al_8_ phase peaks (Figure 4). It was found that the peak intensity of the MoAl_3_ phase decreases as the annealing time is extended (Figure 4b). Conversely, the intensity of the Mo_3_Al_8_ phase peaks increases with increasing annealing time (Figure 4a). This is to be expected because of the extension of the annealing time effect on the diffusion of aluminum, and the formation of phases containing smaller amounts of aluminum. In addition, a shift to the right of the peaks from the molybdenum-aluminum phases was observed, which may be due to the additional silicon content in these phases.

### 3.2. Influence of Slurry Volume on Coating Microstructure

#### 3.2.1. Silicide Coatings

**Figure 5:**
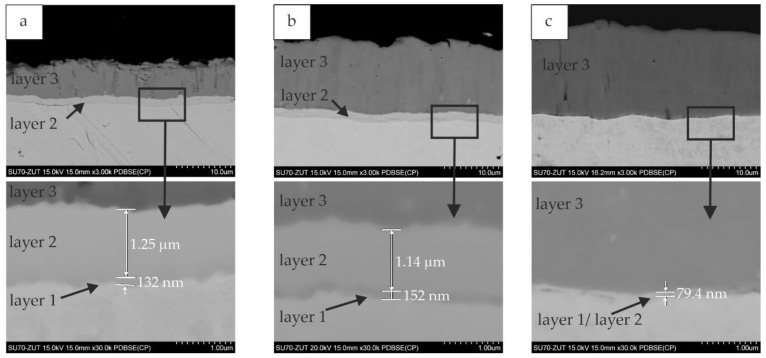
The microstructure (BEI) of Si coatings obtained at 1000 °C for 4 h with the slurry volume of (**a**) 0.15; (**b**) 0.3; and (**c**) 0.6 g/cm^2^.

**Figure 6:**
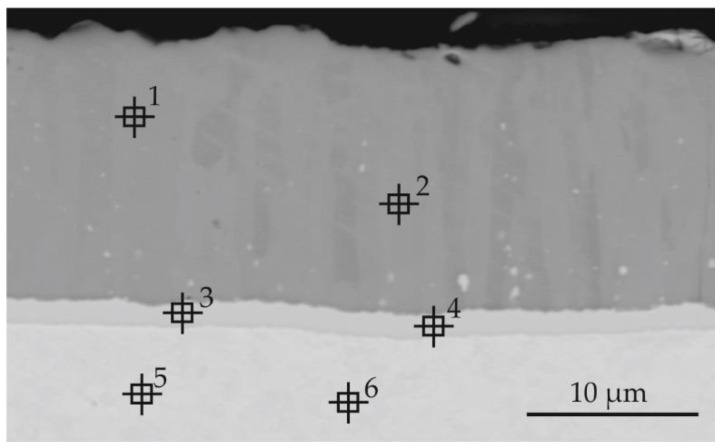
Microstructure (BEI) and point X-ray microanalysis is of Si coating obtained on TZM alloy for 6 h at 1000 °C with the slurry volume of 0.3 g/cm^2^.

Typical microstructures of the silicide coatings obtained for the different slurry volumes are presented in Figure 5. For slurry volumes of 0.15 and 0.3 g/cm^2^ with annealing times of 4 or 6 h, the third sub-layers described previously could be clearly distinguished, see Figure 5a,b. In the case of the coatings obtained with the highest slurry volume, the morphology was slightly different. The cross-sections showed that the bulk of the coating volume was composed of the MoSi_2_ phase (sub-layer 3 in Figure 5c). A thin sub-layer close to the substrate could be distinguished, but it was not possible to establish whether this thin sub-layer contained one or two phases. Only with the coatings obtained after annealing for 6 h was this sub-layer thick enough to carry out microanalysis (Figure 6), which revealed a decreased silicon content in this area (Table 2). However, an accurate phase analysis was not possible. It can be only hypothesized, that also Mo_5_Si_3_ and Mo_3_Si phases were formed in this area, according to the phase diagram [9,26]. There is a difficulty in observing and distinguishing between individual layers of coatings produced under certain conditions. If three silicide phases grow simultaneously, the coatings consisted of the Mo_5_Si_3_ phase always contain the Mo_3_Si phase as well.

The silicon atoms from the slurry are deposited onto the molybdenum alloy surface, where a chemical reaction occurs to form the MoSi_2_ phase. Over time, silicon is released by a decomposition reaction of the MoSi_2_ phase into the Mo_5_Si_3_ phase and silicon. This silicon diffuses into the substrate, and at the same time, molybdenum diffuses from the substrate towards the surface. However, silicon is the dominant diffusing element in all of the Mo–Si phases (MoSi_2,_ Mo_5_Si_3_ and Mo_3_Si). The diffusion of silicon is appreciably larger than that of molybdenum in the MoSi_2_ phase and also larger in the Mo_5_Si_3_ and Mo_3_Si phases [9,28]. In the MoSi_2_ phase, Mo is practically immobile [28]. Thus, providing a large amount of silicon from the slurry causes a continuous increase in the MoSi_2_ layer, whilst if only a small amount of silicon is available in the slurry, this should result in the Mo_5_Si_3_ phase forming from the MoSi_2_ phase and further silicon diffusion inwards. 

A smaller amount of silicon in the slurry and an extended process time may result in the appearance of Kirkendall voids [9,25,29], resulting from the different diffusion coefficients of silicon and molybdenum. The absence of Kirkendall voids in the present studies is observed. At this temperature (1000 °C), the amount of silicon from the slurry is expected to be sufficient to prevent the formation of Kirkendall voids. In previous work, Kirkendall voids were observed by the authors only in silicide coatings manufactured at 1200 °C [22]. Studies [30] have shown that, in well-annealed MoSi_2_, structural vacancies do not exist.

The MoSi_2_ layer has a columnar microstructure (Figure 6), with grains exhibiting a preferred direction of growth parallel to the diffusion direction [6,9,12]. The diffusion anisotropy in MoSi_2_ is a consequence of its tetragonal C11_b_ structure [31]. The diffusion coefficient of a tetragonal crystal, such as α-MoSi_2_, has two principal components—perpendicular and parallel to the tetragonal axis. The silicon diffusion perpendicular to the tetragonal axis is faster than parallel to it.

#### 3.2.2. Silicide-Aluminide Coatings

**Figure 7:**
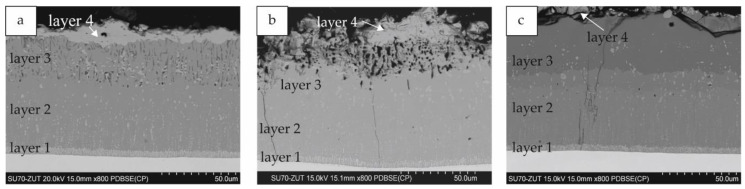
The microstructure (BEI) of Si-Al coatings obtained at 1000 °C for 4 h with the slurry volume of (**a**) 0.15; (**b**) 0.3; and (**c**) 0.6 g/cm^2^.

The microstructure of the silicide-aluminide coatings was much more diverse than that of the silicide coatings and depended on the slurry volume used in the experiments. Example cross-sections are presented in Figure 7. All sub-layers 1–4 mentioned in Section 3.1 were identified in the coatings obtained with each slurry volume (0.15, 0.3, and 0.6 g/cm^2^) used in the current study.

The first layer closest to the substrate was characterized by the smallest thickness and a compact columnar structure. The second layer was characterized by a compact structure without voids with small precipitations throughout its cross-section. Sub-layer 3 was characterized by a certain porosity, and for some coatings, it had also higher aluminum content than sub-layer 2. The development of this sub-layer 3 was different depending on the time of annealing and the volume of the slurry used during the process.

**Figure 8:**
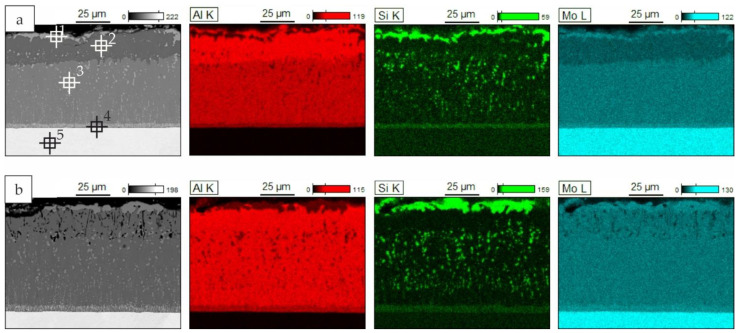
Elemental mapping (BEI/EDS) of cross–sections of Si-Al coatings obtained on TZM alloy at 1000 °C with the slurry volume of 0.15 g/cm^2^ for (**a**) 2; (**b**) 4 h (with points of X-ray microanalysis).

**Figure 9:**
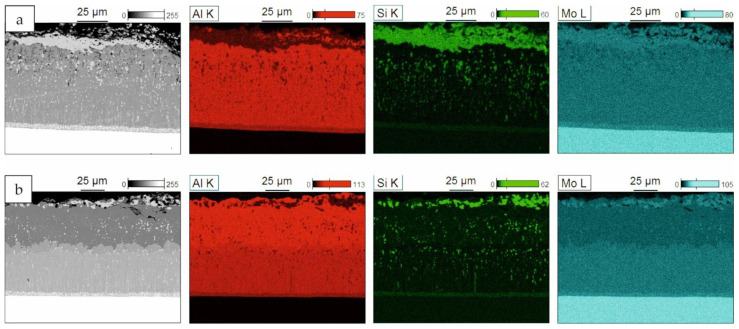
Elemental mapping (BEI/EDS) of cross-sections of Si-Al coatings obtained on TZM alloy at 1000 °C with the slurry volume of (**a**) 0.3 g/cm^2^ and (**b**) 0.6 g/cm^2^ for 6 h.

Elemental maps for the coatings obtained with the smallest slurry volume after 2 and 4 h of annealing are presented in Figure 8. The coatings obtained after 6 h had the same elemental distribution as the coatings after 4 h of annealing. It can be observed that, after 2 h, sub-layer 3 was richer in aluminum than sub-layer 2 (MoAl_3_—Figure 8a, point 2, Table 3). Also, the silicon content was slightly higher in this sub-layer. After 4 and 6 h of annealing, the difference in aluminum content was insignificant; however, numerous voids were present in the area of sub-layer 3. This is probably due to an insufficient supply of aluminum. The elemental maps for coatings obtained with 0.3 and 0.6 g/cm^2^ of the slurry after 6 h are presented in Figure 9. For the coatings obtained with the highest slurry volume, a clear sub-layer 3 had already formed after 2 h of annealing and remained unchanged after 4 h (Figure 7c). This layer had a high porosity and a higher aluminum content than sub-layer 2. After 6 h of annealing, the porosity was not observable, but there was still an observable difference in aluminum content between these sub-layers (Figure 9b).

In general, the largest number of voids in layer 3 was observed with less slurry volume and longer time of annealing; in coatings prepared with 0.15 and 0.3 slurry after annealing for 4 and 6 h and for 6 h, respectively.

The growth of the coatings by solid-state diffusion of Al and Si to the substrate could be dependent on the properties of these elements. The atomic size of silicon (1.32 Å) is smaller than that of aluminum (1.43 Å) [32]. Also, valence state of silicon (4) is higher than aluminum (3). This means that silicon should diffuse faster than aluminum. The atomic size of molybdenum is between that of aluminum and silicon and amounts to 1.39 Å. Diffusion rate is dependent on molecular size because larger molecules diffuse slower than smaller molecules. Because diffusion relies on movement and movement relies on the size of the molecule, there is a direct relationship between molecular size and diffusion rate. Larger molecules necessitate larger amounts of energy to engage in the same level of activity as smaller molecules.

Summarizing, for smaller slurry volume and longer annealing time, the formation of voids may be the result of a depletion of the elements from the slurry and a simultaneous inward diffusion of silicon. Increasing the slurry volume, even for a longer annealing time, reduces the formation of voids because the elements are still being supplied from the slurry.

### 3.3. Influence of Slurry Volume on Coating Thickness

#### 3.3.1. Silicide Coatings

**Figure 10:**
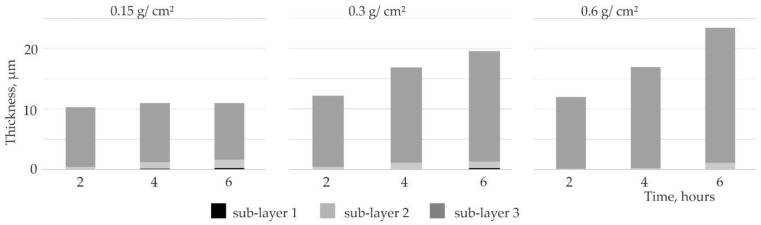
Thickness of Si coatings obtained at 1000 °C for different slurry volume and after varied annealing times.

The thickness of the silicide coatings increased with annealing time (Figure 10). The total thickness of the coatings depended only on the thickness of sub-layer 3. The growth of the silicon-rich sub-layer depended strongly on the slurry volume used in the experiments. The third layer obtained after 2 h of annealing with any slurry volume had a similar thickness. The thickness of the third layer was also similar for coatings manufactured with the 0.15 g/cm^2^ slurry volume, even with the time extension. For slurry volumes of 0.3 and 0.6 g/cm^2^, the third layer obtained after 4 h of annealing were similar, and 50% thicker than the third layer obtained with the smallest slurry volume. The provision of silicon over time increased the thickness of the MoSi_2_ sub-layer.

The thicknesses of sub-layers 1 and 2 also changed depending on the time and the slurry volume. If the amount of silicon was limited (0.15), a more intensive growth of the Mo_5_Si_3_ sub-layer, followed by the MoSi_3_ sub-layer, was observed. This increase of the first and second layers was at the expense of the MoSi_2_ sub-layer.

The rate of silicon diffusion through the obtained silicide coating decreases as the MoSi_2_ sub-layer thickness increases. Further annealing leads to the simultaneous growth of both the Mo_5_Si_3_ and the Mo_3_Si sub-layers. This clearly indicates that the silicon supply was a factor limiting the coating growth.

#### 3.3.2. Silicide-Aluminide Coatings

**Figure 11:**
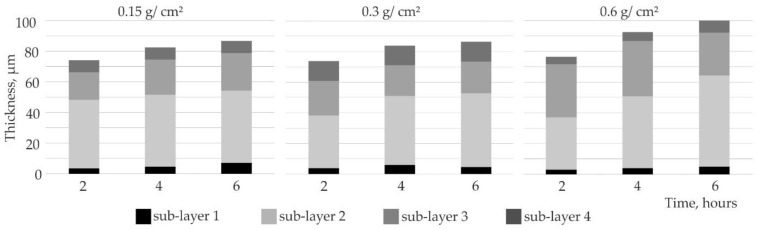
Thickness of Si-Al coatings obtained at 1000 °C for different slurry volume and after varied annealing times.

The thickness of the silicide-aluminide coatings was much larger than the silicide coatings obtained using the same process conditions (Figure 11). After 2 h of annealing, the thickness of all the coatings was similar (approximately 75 µm). Differences were observed after 4 or 6 h of annealing. The increases in coating thickness measured after 4 and 6 h of annealing were greater when the slurry volume was larger. It can be also observed that, in the case of the coatings obtained with the highest slurry volume, sub-layers 2 and 3 (composed of aluminum-rich phase) were the thickest. The thickness of the silicon-rich area (sub-layer 4) was comparable to the thickness of the silicide coatings (previously described in Section 3.3.1) obtained in comparable conditions with 0.15 and 0.3 g/cm^2^ slurry (Figure 10).

The process of saturation with aluminum, or aluminum together with silicon, using slurries is a variant of the liquid-phase aluminizing method, where the applied layer of the slurry limits the liquid-phase volume. The results of saturation, that is, the aluminum content of the coating formed, silicon content, and, hence, hardness, ductility, and other parameters, depend on the thickness of the applied slurry layer, the ratio of aluminum and silicon contents in its active component, diffusion annealing temperature, and time [17]. The liquid phase on the saturated surface ensures favorable conditions for adsorption and diffusion processes. Therefore, the coatings formation from the liquid phase when aluminum is in the slurry causes the coatings to grow faster. On the other hand, the liquid state is not obligatory for diffusion coating formation. A diffusion coating may form at saturation from the solid phase as well. In this case the process is more favorable for the formation of intermetallic phases with high heat of formation. Silicide coatings formed without aluminum in the slurry grow from the solid phase Therefore, the thickness of the silicide-aluminide coatings is larger than silicide coatings.

### 3.4. Structure and Growth Model of Coatings

**Figure 12:**
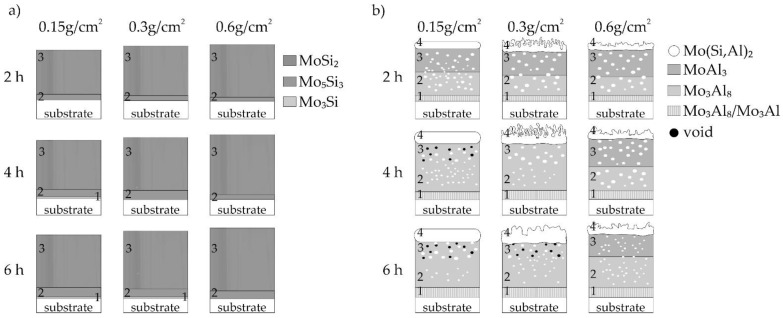
The scheme of the structure of (**a**) Si and (**b**) Si-Al coatings obtained on TZM alloy at 1000 °C for different slurry volume and different time.

The silicide-aluminide coatings were characterized by a more complex structure than the silicide ones. For this reason, a structure diagram of the coatings is presented in Figure 12.

In the case of the silicide coatings, it should be emphasized that they all are very similar in structure (Figure 12a). The outer columnar-structure sub-layer containing the MoSi_2_ phase was dominant in all of these coatings. Coatings produced in 2 h had the same structure, and their thickness increased slightly with increasing volume of the slurry. In coatings produced using a smaller volume of the slurry (0.15 and 0.3), a prolonged annealing time resulted in an increase in the Si-poor first sublayer containing the Mo_3_Si phase.

In the case of the Si-Al coatings, the coating structure observed after annealing for 2 h was similar, regardless of the slurry volume (Figure 12b). Individual sub-layers were different only in thickness (Figure 11). For small volumes of the slurry (0.15 and 0.3 g/cm^2^), an increase in the annealing time caused the MoAl_3_ phase, rich in aluminum, to not occur in the third sub-layer. At the same time, voids were formed in this sublayer. The presence of the Mo(Si,Al)_2_ phase in all coatings was also observed. This phase is of particular importance from the point of view of oxidation stability. In some coatings, the sublayer containing this Mo(Si,Al)_2_ phase was thin (0.15 g/cm^2^) or partially porous (0.3 and 0.6 g/cm^2^). However, oxidation tests are needed in order to verify the high-temperature resistance of the coatings.

Some similarities were observed between the two groups of coatings, both silicide and silicide-aluminide. Coatings produced using the 0.15 g/cm^2^ slurry volume in 4 and 6 h and the 0.3 g/cm^2^ slurry volume in 6 h were very similar for each group of coatings. In addition, the coatings produced in 2 h and using the 0.6 g/cm^2^ slurry volume were also very similar within a given group of coatings.

Additionally, the relations between variable parameters and the thickness of the silicide (1) and silicide-aluminide (2) coatings were determined using Statistica 13 software. The multiple linear regression was used as an approach to modelling the relationship between a dependent variable (the thickness of coatings) and an independent variables (the slurry amount and the time of annealing). The equations thus obtained are presented below. The following notation was used: Y_Si_—the thickness of the silicide coatings (µm); Y_SiAl_—the thickness of the silicide-aluminide coatings (µm); X_1_—the slurry volume (g/cm^2^); X_2_—the annealing time (h).

(1) $$Y_{Si}=5.7+15.1\cdot X_{1}^{2}+1.7\cdot X_{2};R=0.80;\;R^{2}=0.84;F=5.2$$

(2) $$Y_{SiAl}=60.3+35.7\cdot X_{1}^{2}+4.8\cdot X_{2};R=0.91;\;R^{2}=0.84;F=16.3$$

**Figure 13:**
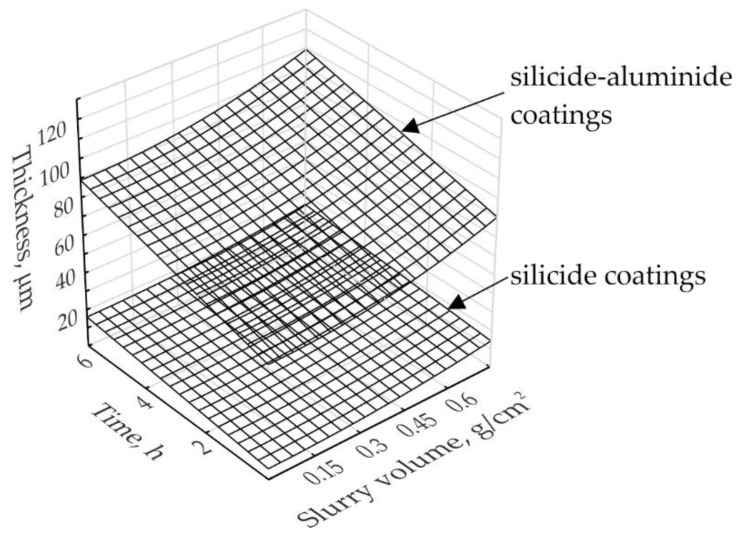
Graphical representation of the correlation between technological parameters and the coatings thickness obtained on TZM alloy at 1000 °C.

The obtained equations indicate the thickness of the coatings produced at the temperature of 1000 °C depends quadratically on the slurry volume parameter and linearly on the time parameter, respectively. The goodness of fit of the obtained models is better for silicon-aluminide coatings (2) than for silicide coatings (1). Graphical representations of the Equations (1) and (2) are shown in Figure 13.

### 3.5. Microhardness of Coatings

**Figure 14:**
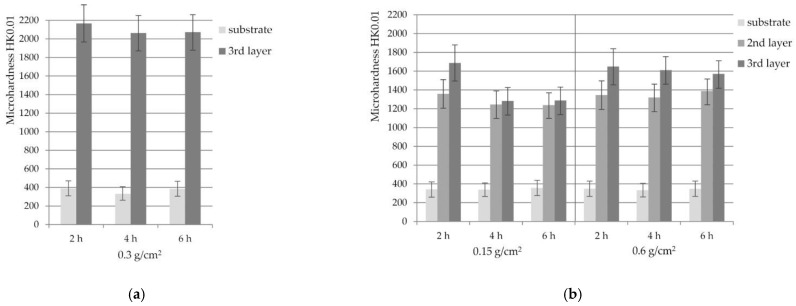
The results of the (**a**) Si and (**b**) Si-Al coating hardness measurements obtained on TZM alloy at 1000 °C for 0.15 and 0.6 g/cm^2^ slurry volume and different time.

Knoop hardness measurements were performed in layers 3 and in layers 3 and 2 for silicide coatings and silicide-aluminide coatings, respectively. Due to the low thickness of the remaining sub-layers, it was not possible to carry out microhardness measurements in these small sub-layers. Additionally, the base material of each sample was also investigated. The figure below (Figure 14) shows the results of microhardness tests for the silicide coatings obtained with the slurry of 0.3 g/cm^2^, and silicide-aluminide coatings with extreme amounts of the slurry 0.15 and 0.6 g/cm^2^.

The microhardness of the external third layer of the silicide coatings (Figure 14a) was about 2100 HK0.01, higher than that of the silicide-aluminide external third layer. The results of the coating microhardness measurements of the silicide coatings were very similar for each coating annealing time due to the same crystalline structure. The average for all silicide coatings was 2096 and 366 for the third layer and substrate, respectively. The standard deviation was 202 and 40 for the above measurements, respectively. The results show that the obtained silicide coatings are very hard and there is a large difference in hardness between the substrate and the coating. This can be the cause of possible cracks during operation, despite the fact that they are diffusion coatings.

In the case of the silicide-aluminide coatings, the microhardness test results (Figure 14b) correspond to the observations of the coating structure. They can be compared with the results shown in Figure 12b. The hardness of the sublayer 3 containing the MoAl_3_ phase is about 1600 HK0.01. This hardness is higher than sublayer 2, containing the Mo_3_Al_8_ phase, which is about 1300 HK0.01. The difference in hardness between sub-layers 2 and 3 is about 300 HK0.01.

Similar hardness values (approx. 1300 HK0.01) were observed for layers 2 and 3 in coatings produced for 4 and 6 h using 0.15 g/cm^2^ of the slurry. The composition of these sub-layers is similar. They contain the Mo_3_Al_8_ phase, which is also shown in Figure 12b.

The resulting coatings with a gradient multi-layer structure were obtained because the aluminum forms several molybdenum aluminides. Each layer of coating contains a different phase component and represents other properties. The gradient of coatings is particularly advantageous in the context of aluminum content. If the aluminum content is greatest in the outer layer, this would promote increased resistance to oxidation or generally high temperature corrosion. Simultaneously, the highest aluminum content decreases the ductility and increases the hardness. Reducing the amount of aluminum in a layer closer to the substrate increases the ductility (decreases the hardness) of the coatings, which reduces the stresses that generate cracks.

## 4. Conclusions

It was demonstrated that the slurry cementation method based on inorganic binder is an effective way to produce both the silicide and the silicide-aluminide coating on molybdenum alloy. There is the ability to easily mix aluminum with other elements (in this study is silicon) that co-spend in the process of coating formation. Compared to other methods, the slurry method is a promising way of producing of the diffusion coatings. 

It was also proven that the slurry volume applied during the slurry cementation of TZM alloy has an important influence on the morphology and growth kinetics of silicide and silicide-aluminide coatings.

The thickness of the sub-layers of the coating can be readily modified by changing the annealing time and the slurry volume. These parameters have a decisive influence on the coating morphology and composition, especially in the case of the silicide-aluminide coatings. Generally, all produced coatings were characterized by a multi-layer structure, silicide coatings—a three-layer structure, and silicide-aluminide coatings—a four-layer structure. The thickness of silicide-aluminide coatings is up to ten times greater than that of silicide coatings produced under the same conditions.

In the case of silicide coatings, it can be stated that:

- the main part of the coating is the outer sublayer containing the MoSi_2_ phase,
- the use of a small volume of the slurry (0.15 g/cm^2^) during production and extension of the annealing time, did not significantly increase the thickness of the coatings, which was about 11 µm. In such conditions, increasing deficiency of the alloying elements slows down the kinetics of the Si-rich layer growth,
- the use of a larger volume of the slurry (0.3 and 0.6) during production and extension of the annealing time, significantly increased the thickness of the coatings, from about 12 µm in the case of 2 h to about 20 µm in the case of 6 h of annealing,
- the hardness of the coatings is similar, around 2100 HK0.01, since all the resulting coatings contain the MoSi_2_ phase.

In the case of silicide-aluminide coatings, the following conclusions can be made:

- the main components of the coatings were the two phases MoAl_3_ and Mo_3_Al_8_, while the outer sublayer contained the Mo(Si,Al)_2_ phase, which should constitute a corrosion barrier,
- the use of a small volume of the slurry (0.15 g/cm^2^) during production and extension of the annealing time resulted in the formation of voids in the cross-section of the coatings, moreover, the formation of phases with lower aluminum content is observed.
- the coating growth kinetics for 0.15 and 0.3 slurry volumes were similar. The thickness of the coatings produced for 2, 4, and 6 h was on average 75, 82, 85 µm, respectively,
- the use of a larger volume of the slurry (0.6) during production and extension of the annealing time, significantly increased the thickness of the coatings, from about 75 µm in the case of 2 h to about 100 µm in the case of 6 h of annealing,
- the hardness of the sublayers increased with increasing aluminum content and ranged from about 1200 to 1700 HK0.01.

## Figures and Tables

**Table 1:** The results of the EDS point analysis of Si-Al coating according to Figure 2.

Point of Analysis	Chemical Composition, % at				
	Al	Si	Mo	Ti	Zr
1	70.3	0.7	29.0	0.0	0.0
2	43.5	14.1	42.4	0.0	0.0
3	19.7	6.5	73.8	0.0	0.0
4	0.7	0.0	99.1	0.2	0.0
5	3.1	0.1	0.3	94.9	1.6
6	0.9	0.3	0.8	95.8	2.2

**Table 2:** The results of the EDS point analysis of Si coating according to Figure 6.

Point of Analysis	Chemical Composition, % at	
	Si	Mo
1	65.2	34.8
2	65.1	34.9
3	45.5	54.5
4	45.5	54.5
5	0.0	100.0
6	0.0	100.0

**Table 3:** The results of the EDS point analysis of Si-Al coating according to Figure 8a.

Point of Analysis	Chemical Composition, % at		
	Al	Si	Mo
1	23.1	42.4	34.5
2	77.0	2.4	20.6
3	70.3	1.1	28.6
4	56.6	7.3	36.1
5	0.8	0.0	99.2

## Data Availability

Data are contained within the article.
